# Clinical Implications of Acquired BRAF Inhibitors Resistance in Melanoma

**DOI:** 10.3390/ijms21249730

**Published:** 2020-12-20

**Authors:** Paola Savoia, Elisa Zavattaro, Ottavio Cremona

**Affiliations:** 1 Department of Health Science, University of Eastern Piedmont, Via Solaroli 17, 28100 Novara, Italy; 2 AOU Maggiore della Carità, c.so Mazzini 18, 28100 Novara, Italy; elisa.zavattaro@med.uniupo.it; 3 Division of Neuroscience, San Raffaele Scientific Institute, Università Vita Salute San Raffaele, Via Olgettina 58, 20132 Milano, Italy; cremona.ottavio@hsr.it

**Keywords:** metastatic melanoma, BRAF inhibitors, target therapy, resistance

## Abstract

Understanding the role of mitogen-activated protein kinase (MAPK) pathway-activating mutations in the development and progression of melanoma and their possible use as therapeutic targets has substantially changed the management of this neoplasm, which, until a few years ago, was burdened by severe mortality. However, the presence of numerous intrinsic and extrinsic mechanisms of resistance to BRAF inhibitors compromises the treatment responses’ effectiveness and durability. The strategy of overcoming these resistances by combination therapy has proved successful, with the additional benefit of reducing side effects derived from paradoxical activation of the MAPK pathway. Furthermore, the use of other highly specific inhibitors, intermittent dosing schedules and the association of combination therapy with immune checkpoint inhibitors are promising new therapeutic strategies. However, numerous issues related to dose, tolerability and administration sequence still need to be clarified, as is to be expected from currently ongoing trials. In this review, we describe the clinical results of using BRAF inhibitors in advanced melanoma, with a keen interest in strategies aimed at overcoming resistance.

## 1. Introduction

Melanoma is the twentieth most common cancer worldwide, with an incidence that has progressively increased over time as a consequence of lifestyles that accentuate photo-induced skin damage [1,2]. However, the mortality rate from this neoplasm is decreasing in Western countries, thanks to efforts that have improved early diagnoses and, above all, to the new acquisitions in the field of metastatic disease treatment. In particular, the landscape of possible therapeutic options for the treatment of advanced disease has been substantially modified by target therapies and by immunological checkpoint inhibitors [3]. However, despite the amazing results obtained with first-generation kinase inhibitors in terms of response rate, the average duration of this response was short due to the onset of resistance to treatment. Combination therapy with BRAF/MEK inhibitors proved to be an excellent way to overcome the resistance mechanisms, such as the recent development of highly specific Extracellular Signal-Regulated Kinase (ERK) inhibitors [4]. Nevertheless, other possible strategies, including the combination of BRAF inhibitors (BRAFi) with immunotherapy, sequencing approaches and rechallenge or retreatment should be considered. In this review, the main clinical implications of BRAFi resistance will be analyzed, along with possible overcoming strategies.

## 2. BRAF Mutations in Melanoma

The mutational burden of melanoma is one of the highest among solid tumors and concerns specific oncogenes involved in cell proliferation and migration. In particular, activating mutations of the MAPK/ERK signaling pathway are the key for melanoma development and progression and are typically associated with a worse prognosis [5]. The activation of this pathway, in fact, promotes cell detachment from the extracellular matrix and cellular motility and activates a number of transcription factors and nuclear proteins implicated in cell cycle regulation [6]. 

BRAF mutations are present in approximately 60% of melanomas, with a variability established by histologic subtype, anatomical location, sun exposure pattern, ethnicity and geographical provenience. The higher mutation rate is observed for superficial spreading and nodular melanoma (50% and 43%, respectively) and in melanomas located in anatomical areas subjected to intermittent sun exposure. On the contrary, BRAF mutations are rarely identified in acral lentiginous and mucosal melanomas [7,8]. The role of BRAF mutations as an independent adverse prognostic factor is proven [9], whereas still debated is the correlation between BRAF mutation, higher Breslow thickness and higher Clark level [10,11,12].

The most frequently observed BRAF mutation is the V600E, resulting in an amino acid substitution of valine (V) with a glutamic acid (E) at position 600 in the BRAF protein as a consequence of the transversion c.1799T>A in exon 15 [13]. This mutation increases the BRAF kinase activity by about 700-fold over wild-type, but strongly active mutants also arise from the other mutations V600K (7.7%), V600R (1%), V600M (0.3%) and V600D (0.1%) [13]. 

## 3. BRAF Inhibitors in Melanoma

The identification of BRAFV600 mutations directed the research towards the production of specific inhibitors of this protein isoform with potential therapeutic value. Among the small molecules that had demonstrated activity in preclinical studies, vemurafenib was the first-in-class BRAF mutated inhibitor approved by the FDA (July 2011) and the EMA (February 2012) for the treatment of unresectable or metastatic melanoma, based on the results from the pivotal study BRIM-3 (NCT01006980) [14]. This was a randomized, open-label, multicenter study comparing vemurafenib monotherapy with dacarbazine (DTIC) that enrolled 675 patients. The difference in overall survival (OS) between the two treatment arms was so significant already in the interim analysis as to allow cross-over. Despite this, vemurafenib reached a very high significance for OS and progression-free survival (PFS) (13.2 months vs. 9.6, and 6.9 months vs. 1.6, respectively), with an overall response rate (ORR)of 48.4% in patients treated with vemurafenib and of 5.5% in those treated with DTIC.

Equally, in 2013, the FDA and the EMA approved the type-I kinase inhibitor of the BRAFV600E/K/D-mutated protein dabrafenib for the treatment of advanced-stage melanoma, based on the results obtained by the multicenter, open-label, phase 3 randomized BREAK-3 (NCT01227889) controlled trial [15]. In addition, for this drug, comparison with DTIC showed a significant improvement of PFS in patients treated with dabrafenib (5.1 months vs. 2.7 months). Moreover, the noncomparative phase II trial BREAK-MB (NCT01266967) also demonstrates a response to dabrafenib in patients with brain metastases [16]. 

However, despite the amazing short-term benefit in terms of ORR, PFS and OS obtained with all BRAFi, the long-term survival was unsatisfying. In fact, in the BRIM 3 study, the two-year survival rate was 17% in the vemurafenib arm vs. 15.6% for DTIC [14]. In addition, three-year and four-year survival rates of 26% and 19%, respectively, were observed in the extension cohort described by Puzanov et al. [17]. Moreover, the two-year follow-up of the BREAK-3 study [15] did not show a significant difference in OS between patients treated with dabrafenib and those who received DTIC. Therefore, encorafenib, a second-generation type-I, ATP-competitive, selective kinase inhibitor, which specifically targets BRAFV600E and BRAFV600K [18], has been approved by the EMA and the FDA exclusively as a combination therapy with the MEKi binimetinib, despite an ORR of 60% achieved as a single agent in BRAFi-naïve patients [19].

## 4. Resistance to BRAF Inhibitors 

Different resistance mechanisms to BRAFi have been described. These can be categorized as (i) primitive (intrinsic) resistance, which confers an innate resistance to the treatment, and (ii) secondary (extrinsic) resistance, which determines a progressive loss of efficacy during treatment. 

### 4.1. Intrinsic Resistance

A high percentage of melanoma patients do not respond to BRAFi therapy, although they carry an activating BRAF mutation; these patients are defined as primarily, intrinsically or innately resistant to targeted therapies aimed at downregulating or suppressing the MAPK signaling pathway. A first estimate of the percentage of BRAFi-unresponsive patients with BRAF-mutated melanoma came from the BRIM2 phase 2 clinical study [20], in which an impressive unresponsive rate of 47% to vemurafenib monotherapy was observed that was then confirmed by the phase-3 study [14]. Among the intrinsic resistance mechanisms, mutations of several genes, including RTK, COT, NF1, RAC1, PTEN and CDKN2A/CDK4, can increase melanocytes’ survival and promote their proliferation and migration, resulting in a reduced sensitivity to BRAFi [21,22]. 

Moreover, the involvement of the tumor microenvironment should be considered. Recently, it has been demonstrated [23] that the stromal secretion of Hepatocyte Growth Factor (HGF) leads to MAPK and PI3K pathway activation, with a consequent resistance to BRAF inhibition. Furthermore, an immune-mediated mechanism of resistance has been hypothesized; despite the initial favorable effects on the tumor microenvironment exerted by BRAFi and MEKi, low levels of CD8+ tumor-infiltrating T cells were observed in patients who rapidly progressed under targeted therapy [24], together with an increased expression of the immune inhibitory molecule programmed death-ligand PD-L1 [25].

### 4.2. Extrinsic Resistance

The acquired resistance to BRAFi through the reactivation of the MAPK pathway, occurring upstream, downstream or at the BRAF level, plays a crucial role in the majority of patients affected by advanced melanoma due to the relatively short PFS observed when treated with BRAFi alone. 

One of the most studied mechanisms is represented by the presence of activated forms of RAS, which, in melanoma cell lines and transgenic mice, are associated with a BRAFi resistance [26,27]. In a clinical setting, NRAS mutations have been identified in 8–20% of patients that developed resistance after vemurafenib or dabrafenib treatment [28,29]. In addition, in melanoma BRAF^mut^ cells, resistance has been found to be associated with high CRAF levels and ectopic CRAF expression [30,31]. 

Activating MEK1 mutations are reported in 7% of progressed patients [29]; this type of mutation confers resistance not only to BRAFi but also to MEKi, resulting in reduced effectiveness of the entire combination therapy, while sensitivity to ERK inhibitors is broadly maintained.

Splice variants or amplifications of the BRAF gene have been described in a percentage of patients, ranging from 16% to 32% and 8% to 12%, respectively [28,29]. Recently, the acquisition of a secondary BRAF^L514V^ mutation has been identified as a further possible mechanism responsible for the acquired BRAFi resistance in a BRAF^V600E^ mutant patient affected by a brain tumor, who experienced a complete response followed by a recurrence under dabrafenib treatment [32]. In addition, BRAF^L505H^ has been identified as a secondary mutation associated with acquired resistance to vemurafenib in melanoma [33]. However, combination therapy with BRAFi/MEKi and the possible association with ERKi should allow these resistance mechanisms to be overcome. The role of other mutations affecting several genes, including ERK1/2, RTK, MITF, PTEN and PI3K/AKT, is also conceivable for the onset of the acquired resistance [34]. Notably, ERK inhibition leads to MITF gene amplification and, therefore, to an increase of mitochondrial activity and cellular metabolism, with greater chances of survival for cancer cells. 

Specific associations between the acquired mutation pattern and the clinical course have been identified; in particular, NRAS mutations occur most commonly in patients treated with vemurafenib alone and are predominantly implicated in intracranial progression [35]; a high rate of BRAF secondary mutation was found in brain metastasis [36], whereas MEK1/2 mutations are associated with the development of hepatic metastases [29]. However, intertumoral and intratumoral heterogenicity has been demonstrated by several authors; in a recent study [37], Chang et al. confirmed a mutational heterogeneity regarding the BRAF, NRAS and TERT genes in 18% of patients, but data obtained by different groups report variable frequencies. Similarly, two or more concomitant resistance mechanisms have been found in 20% of patients analyzed by Gowrishankar et al. [38]. 

## 5. Beyond the Resistance

### 5.1. BRAF/MEK Inhibitors

As mentioned above, in the majority of advanced melanomas, the main resistance mechanism to BRAFi is due to the paradoxical reactivation of the MAPK pathway [39]. As a consequence, the most intuitive strategy to overcome resistance is represented by the association of BRAFi with other kinase inhibitors downstream in the MAPK phosphorylation pathway, i.e., MEKi/ERKi. This combination provides a number of additional advantages, including a more potent and longer-lasting clinical response [40,41,42,43] and reduced toxicity in comparison with BRAF inhibitor monotherapy; it has also been shown that the MEKi/ERKi combination is effective in the retreatment of patients who have developed resistance to BRAFi alone [44].

#### 5.1.1. Trametinib

Following the encouraging results obtained by the COMBI-D phase III trial (NCT01584648) [41], the combination therapy dabrafenib/trametinib was approved in August 2013 by the EMA and in January 2014 by the FDA. The COMBI-D trial showed that the advantages of the combination were not only represented by higher PFS and OR rates in patients treated with BRAFi plus MEKi than in those treated with dabrafenib alone (9.3 vs. 8.8 months and 67% vs. 51% respectively) but also by lower toxicity in the combination therapy group compared with BRAFi alone, as a consequence of the inhibition of the paradoxical activation of ERK, which is responsible for most of the adverse events (AEs) observed during the administration of BRAFi alone. In fact, the overall percentage of patients who developed AEs of any grade was 91% in the combination therapy group and 98% in the vemurafenib group, but the percentage of grade 3–4 AEs was 52% and 63%, respectively. These results have been confirmed also in long-term overall survival analyses [45,46]. The three-year OS and PFS were 44% and 22%, respectively, in patients who received the combination and 32% and 12%, respectively, in patients treated with the BRAF inhibitor alone, with an OS that reached 62% in the most prognostically favorable subgroups [45]. Moreover, the pooled extended-survival analysis of patients included in the COMBI-D and COMBI-V trials demonstrates a PFS of 21% at four years and 19% at five years, with overall survival rates of 37% at four years and 34% at five years [46]. These outcomes were highest in patients who achieved a complete response (CR) of 19%, with a five-year ORR of 71%. A safety profile consistent with those previously described was also proved. 

The effectiveness of this combination has been confirmed in the adjuvant setting by the COMBI-AD study [47], in which were included 870 stage IIIA, B and C patients, who were randomized versus double placebo. After a median follow-up of 2.8 years, the estimated three-year rate of relapse-free survival was 58% in the combination therapy and 39% in the placebo groups, with three-year OS rates of 86% and 77%, respectively. The recently published [48] five-year analysis showed 52% and 36% of patients alive without relapse in the combination therapy and the placebo groups, respectively. 

#### 5.1.2. Cobimetinib

At the end of 2015, based on the result of the co-BRIM phase III clinical trial (NCT01689519) [49], the EMA and the FDA reached approval of MEKi cobimetinib for the treatment of unresectable or metastatic melanoma, in combination with vemurafenib. This clinical trial enrolled 495 patients, who were randomized to receive vemurafenib and cobimetinib or vemurafenib and a placebo. The combination group showed a response rate (RR) and a PFS significantly higher than the placebo group (68% vs. 45% and 9.9 vs. 6.2 months, respectively); moreover, a 10% rate of CR was achieved in patients treated with the combination vs. 4% in the control group. These clinical benefits have been confirmed by the updated efficacy analysis in which the median OS was 22.3 months in the combination group, without significant differences in high-grade AEs [50].

At present, no direct comparison between the different BRAFi/MEKi combinations in metastatic melanoma has been performed. However, in 2017, Daud et al. published an indirect comparison between the co-BRIM (vemurafenib plus cobimetinib vs. vemurafenib) [51] and the COMBIv (dabrafenib plus tramentinib versus vemurafenib) [52] clinical trials, analyzing the safety and the OS, PFS and ORR outcomes. This indirect comparison did not show significant differences between the two combinations concerning the various outcomes; however, serious adverse events occurred less frequently in the dabrafenib/trametinib combination group. More recently, Consoli et al. [53] also extended the indirect comparison to the Columbus clinical trial (encorafenib plus binimetinib) with an adjusted meta-analysis that included a total of 1230 patients. In addition, this study did not show significant differences across the different trials in OS, PFS and ORR, whereas a slight difference in the safety profile of the three drug couples was related to their specific pharmacological properties.

#### 5.1.3. Bimetinib

Binimetinib is a selective MEK1 and 2 inhibitor, the clinical activity of which has been confirmed in a phase II trial (NCT01320085) [54] that enrolled 185 patients affected by unresectable locally advanced or metastatic melanoma harboring BRAFV600 or NRAS mutations. A partial response was demonstrated in 20% of BRAF_mut_ patients; the percentage of responders was similar also in the RAS_mut_ subgroup, with a fair safety profile. Later, the effectiveness of binimetinib on RAS_mut_ melanoma patients was evaluated in comparison with dacarbazine in the phase III NEMO trial (NCT01763164) [55], which enrolled 402 patients previously untreated or progressed after immunotherapy. The median PFS obtained with binimetinib was higher than that obtained with dacarbazine (2.8 vs. 1.5 months, respectively), even without significant improvement in the OS.

The combination of binimetinib with BRAFi encorafenib was assessed in the phase III COLUMBUS trial (NCT01909453) in which patients were randomly assigned (1:1:1) to receive encorafenib (300 mg daily), encorafenib plus binimetinib (450 mg plus 90 mg daily) or vemurafenib (960 mg twice daily) [56,57]. Both encorafenib plus binimetinib and encorafenib monotherapy demonstrated the highest efficacy compared with vemurafenib, with a median PFS of 14.9 months in the encorafenib plus binimetinib group, 9.6 months in the encorafenib group and 7.3 months in the vemurafenib group; as expected, the safety profile was more suitable in patients treated with the combination [56]. These data were also confirmed after a four-year follow-up [58].

The potential effectiveness of the combination of encorafenib/binimetinib with other classes of targeted drugs, such as CDK4/6, FGFR, c-Met and PI3K inhibitors, is being studied in the currently ongoing LOGIC-2 trial (NCT02159066). To date, no results have been released for this study, estimated primary completion date of which is January 2022. 

### 5.2. Other Combination Therapies

The capability of BRAFi and MEKi to interact with the tumor microenvironment and their possible immunomodulatory role constitutes the rationale for their combination with immunological checkpoint inhibitors, and, recently, several trials were designed with the aim to verify the efficacy and safety of the triple combination. This approach is supported by several preclinical and translational studies showing the immunological effects of BRAFi/MEKi. In particular, BRAFi are able to increase the intratumoral T-cell infiltrate and upregulate the expression of several melanoma differentiation antigens (i.e., MART-1, gp100 and TYRP-1 and -2) and of major histocompatibility complex MHC type I and II antigens [59]. Conversely, MEKi protect the tumor-infiltrating CD8+ T cell from death [60]. On the other hand, it has been shown that PDL1 status, as well as the density of the intratumoral infiltrate, may have a predictive role for estimating the onset of resistance in patients treated with BRAFi [61].

The effectiveness of the triple combination of dabrafenib, trametinib and pembrolizumab has been investigated in the phase I/II KEYNOTE-022 trial (NCT02130466) that enrolled previously untreated BRAF-mutated advanced melanoma patients. The ORR achieved was 67% in phase I [62] and 63% in phase II of the study [63], with a better median PFS (16.0 vs. 10.3 months) in patients who received pembrolizumab. In addition, 68 patients were enrolled in a non-randomized clinical trial (NCT02027961) that evaluated the combination of dabrafenib/trametinib plus the anti-PD-L1 antibody durvalumab; BRAF_mut_ patients received the triple combination concomitantly, whereas BRAF_wild-type_ patients were treated with trametinib and durvalumab concomitantly or sequentially. The ORR was 76% in BRAF mutated and 21% and 50%, respectively, in BRAF wild type patients treated with trametinib and durvalumab concomitantly or sequentially [64]. Moreover, patients included in the phase III COMBI-I study and treated with the triple combination dabrafenib/trametinib plus the anti PD-1 antibody spartalizumab showed an ORR of 78%, with 44% of CR [65]. The triple combination BRAFi/MEKi/anti-PD1 is being evaluated also in the NCT02130466 and NCT02224781 clinical trials, for which the estimated completion dates are expected to be July 2021 and October 2022, respectively.

The triple combination of vemurafenib, cobimetinib and atezolizumab (a fully humanized antibody against PD-L1) gave encouraging results in the Phase Ib study NCT01656642, which enrolled 67 patients affected by metastatic or unresectable stage III melanoma and achieved a 71.8% ORR, with an estimated median duration of response of 14.4 months [66]. These results were confirmed by the phase III IMspire150 trial (NCT02908672), in which the triple combination was compared to vemurafenib/cobimetinib/placebo on 514 patients randomly assigned to the two groups [67]. In this study, the PFS was significantly higher in patients treated with the triple combination than in the control group (15.1 vs. 18.6 months, respectively, *p* = 0.0025), without significant differences in treatment-related adverse-events. 

The first report from the ongoing SECOMBIT trial (NCT02631447), a phase II study evaluating the best sequencing approach with the combination of encorafenib plus binimetinib with ipilimumab plus Nivolumab have been recently released [68]. At a minimum follow up of one year, the median PFS was 15.8 months for patients who received targeted therapy until progressive disease (PD), followed by ipilimumab plus nivolumab; for patients treated with the reverse combination, the median PFE was 7.2 months, whereas, for arm C, who received targeted therapy for eight weeks, followed by ipilimumab plus nivolumab until PD, followed by targeted therapy, the median PFS was 11.4 months. In fact, the two-year PFS rate is similar among the different arms. For this study, the estimated primary completion date is April 2021. Moreover, in October 2018, the randomized comparative phase II EBIN study (NCT03235245) was started with the objective of evaluating the impact on the PFS of a sequential approach with encorafenib plus binimetinib administered for 12 weeks and followed by combination immunotherapy with nivolumab plus ipilimumab. For this study, the estimated number of patients enrolled is 270 and the estimated primary completion date is February 2024. Encouraging data also derive from real life experiences; a recently published case report demonstrated a clinical response in a patient treated with an unconventional timeline of target and immunotherapy that allowed treatment-related resistances to be overcome [69]. 

The combination of BRAFi/MEKi and immune checkpoint inhibitors has also been proposed as a rescue in patients who progressed under immunotherapy. A recent multicenter study enrolled 61 patients with progressive disease after treatment with anti-PD1 or anti-CTLA4 [70]. BRAF_mut_ patients received the anti-PD1 together with BRAFi and/or MEKi, whereas BRAF_wild-type_ received the anti-PD1 plus MEKi combination. The ORR was 12% and 11%, respectively, with a disease control of 52% and 83% and a median OS of eight and 10.2 months, respectively, for BRAF_mut_ and BRAF_wild-type_ patients.

Less encouraging were the results obtained from the trials that evaluated the possible combination of BRAFi with high-dose IL-2. The multi-center phase II NCT01683188 clinical trial enrolled 53 BRAFmut patients, who received vemurafenib followed by high-dose IL-2, obtaining an ORR similar to that observed with high-dose IL-2 alone (a three-year survival rate of 27–30%) [71]. Higher ORR (83.3%) was obtained in the six patients enrolled in the NCT01754376 clinical trial, who received two courses of high-dose IL-2 together with vemurafenib, with a median PFS of 35.8 weeks. The possible synergistic effect of the two drugs was, however, hindered by the increase in T regulatory cells in the peritumoral infiltrate that was induced by IL-2 in all patients [72]. Currently, other phase I/II clinical trials (NCT01943422; NCT01959633; NCT01603212; NCT01659151) are ongoing, evaluating the possible combination of vemurafenib with IFN and/or IL-2. Moreover, a recently published study [73] demonstrated that pre-treatment with temozolomide, vincristine, lomustine and IFN-alpha-2a followed by vemurafenib significantly increased ORR, PFS and OS with an acceptable safety profile, which, however, required a dose reduction in vemurafenib.

Indeed, the issue regarding combo-immunotherapy toxicity has yet to be fully resolved. A phase I study evaluating the concurrent administration of vemurafenib and ipilimumab [74] was interrupted due to the high-grade liver toxicity observed, and a possible increased risk of colitis in patients who received dabrafenib and trametinib followed by ipilimumab was suggested [75]. In addition, 73% of patients enrolled in KEYNOTE-022 experienced grade 3–4 treatment-related AErs, with dose-limiting toxicities of 20% [63], and grade 3–4 AErs were observed in the 72% of patients enrolled in the COMBI-I trial, with permanent treatment discontinuation in 17% [76].

### 5.3. Rechallenge, Retreatment and Intermittent Treatment 

Rechallenge with target therapy has been proposed as a possible strategy for overcoming resistance in numerous malignancies, including lung and renal cancer and gastrointestinal stromal tumors [77,78,79], as well as in melanoma [80]. A recent multicenter study involving 167 patients, who were rechallenged with BRAFi and MEKi after a disease progression, demonstrated a RR of 37.3% [81]. A slightly lower (32%) but still relevant RR was demonstrated in a similar study involving 25 patients with progressive disease after BRAFi/MEKi treatment [82]. In these patients, a 12-week period of treatment with immunotherapy preceded the rechallenge. However, the RR exceeds that obtainable with immunotherapy alone, suggesting the possible overcoming of the previously acquired resistance to BRAFi. More recently, a multicenter retrospective study [83] evaluated 51 patients with progressive disease after first line of treatment with BRAFi/MEKi and second line immunotherapy with anti PD1/PDL1 or anti CTLA4. Patients were rechallenged with BRAFi/MEKi, with an overall response rate of 27%. Interestingly, the time interval between the end of the first BRAFi/MEKi treatment and the beginning of rechallenge did not influence overall and progression-free survival. On the other hand, the possibility of responding to the rechallenge with BRAFi/MEKi alone had been previously demonstrated in individual case reports or small series [84,85,86,87,88,89]. In particular, Seghers et al. [84] reported a marked clinical response to rechallenge in two patients with documented progression during dabrafenib plus trametininb treatment, after treatment-free intervals of eight and four months, respectively. Moreover, Vanhaecke et al. [88] confirmed the effectiveness of the rechallenge on 16 patients, with a documented response or a disease stabilization in the 62% of the patients retreated with the combination of BRAFi and MEKi following previous treatment with BRAFi alone, after a median interruption of 12 months. 

Long-term maintenance of the previously obtained response has also been reported for patients treated with vemurafenib with an intermittent dosing schedule, with lower toxicity than under continuous administration [86,87,90]. More recently, a long-term (more than three years) response was also described in two cases of NRAS_mut_ metastatic melanoma treated with binimetinib with an intermittent schedule (three weeks on/10-days-off or two weeks on/one week off) [91]. However, the recently published [92] data from a phase II clinical trial enrolling 206 patients randomized 1:1 to receive continuous or intermittent treatment with dabrafenib plus trametinib did not demonstrate a benefit with this latter dosing schedule. The median progression-free survival was 9.0 months in patients who received continuous treatment versus 5.5 months in those treated with the intermittent dosing, without differences in the overall survival and the incidence of treatment-related AErs between the two groups.

### 5.4. New Perspectives 

Based on the results obtained by preclinical and clinical studies [93,94], it was hypothesized that vorinostat, an HDAC inhibitor approved by the FDA for the treatment of progressive, persistent or recurrent cutaneous T-cell lymphoma patients, can remove cell clones that have acquired resistance after treatment with BRAFi/MEKi by remodeling the expression of MITF. In 2016, a small clinical trial (NCT02836548) was drawn [95], with the aim of demonstrating the efficacy of sequential treatment with vorinostat and BRAFi/MEKi in resistant BRAFV600 mutant melanoma. After a wash-out period of three days, the study plans to administer vorinostat 360 mg for 14 days and then to reintroduce BRAFi/MEKi treatment on day 15. At present, results from this very promising study are still waiting to be published.

More recently, the discovery that the majority of BRAFi resistance pathways are directed towards the activation of transcription factor c-MYC pioneered the use of small molecules such as JQ1 in overcoming the acquired resistance to BRAFi [96,97]. In addition, significant detectable levels of Urokinase Plasminogen Activator Receptor (UPAR) and Epidermal Growth Factor Receptor (EGFR) were found in tumor biopsies of who patients who relapsed after vemurafenib treatment [98]. Targeting these molecules with an integrin antagonist peptide, the responsiveness to vemurafenib was restored with a potential therapeutic value. 

Furthermore, based on in vitro studies [99], therapeutic regimens combining BRAFi with bevacizumab or with inhibitors of the PI3K/AKT/mTOR pathway were proposed for overcoming BRAFi resistance. However, the efficacy and tolerability of these possible associations still need to be verified. The phase I/II clinical trial NCT01512251 that combined vemurafenib with the PI3K inhibitor buparlisib (BKM120) was terminated early due to dose limiting toxicities. At present, no data are available from the phase I/II clinical trial NCT01616199, which enrolled 24 patients, randomized to receive the PI3K inhibitor PX-866 in combination with vemurafenib. 

## 6. Conclusions

The low impact of standard chemotherapy on the survival of advanced-stage melanoma patients has been largely overcome thanks to the possibility of using small molecules targeting the MAPK pathway. However, intrinsic resistances to MAPK inhibitors and, even more importantly, the acquisition of resistances during treatment through extrinsic mechanisms limit the response sizes and duration. The synergistic use of several inhibitors blocking the pathway at different points has been shown to be promising, as has the association with immunological checkpoint inhibitors. Indeed, the ongoing studies’ results and the new trials’ designs are essential for identifying the most effective treatment schedules and better managing treatment-related toxicities.

## Abbreviations

AEs	Adverse Events
BRAFi	BRAF inhibitor
BRAF_mut_	BRAF mutated
CR	Complete Response
DTIC	Dacarbazine
EGFR	Epidermal Growth Factor Receptor
EMA	European Medicine Agency
ERK	Extracellular Signal-Regulated Kinase
FDA	Food and Drug Administration
HDAC	Hystone Deacetylase
HGF	Hepatocyte Growth Factor
IFN	Interferon
MHC	Major Histocompatibility Complex
MAPK	Mitogen-Activated Protein Kinase
MEKi	MEK inhibitor
NRAS_mut_	NRAS mutated
ORR	Overall Response Rate
OS	Overall Survival
PFS	Progression-free Survival
RR	Response Rate
UPAR	Urokinase Plasminogen Activator Receptor
